# Acute respiratory syndrome following accidental inhalation of mercury vapor

**DOI:** 10.1002/ccr3.1656

**Published:** 2018-06-21

**Authors:** Jorge Cortes, Jaime Peralta, Rienzi Díaz‐Navarro

**Affiliations:** ^1^ Departamento de Medicina Interna Universidad de Valparaíso Viña del Mar Chile

**Keywords:** acute mercury inhalation, elemental mercury, mercury toxicity, mercury vapor

## Abstract

Although the manufacture of glass mercury thermometers is now prohibited, they are still present in daily life and represent a source of accidental mercury intoxication. Physicians should be able to recognize the clinical manifestations of mercury poisoning caused by accidental exposure and know the appropriate treatment for this toxicological emergency.

## 1 INTRODUCTION

Although rare, mercury vapor inhalation is always a toxicological emergency.^1^ Mercury is a heavy metal that is distributed in the environment as elemental mercury, organic mercury, and inorganic mercury.^2^ Some home sources of exposure to elemental mercury in humans are thermometers, sphygmomanometers, and barometers.^3^, ^4^ The severity of mercury poisoning depends on the concentration at the time of exposure, route, and duration of the exposure.^5^

At room temperature, mercury is highly volatile and can vaporize,^1^ and its volatility increases with heat.^3^, ^6^ Inhalation of mercury vapor causes chemical pneumonia that can evolve to respiratory distress, respiratory failure, and death.^7^, ^8^

We report a case of nonfatal acute respiratory syndrome following accidental inhalation of elemental mercury vapor.

## 2 CASE REPORT

A 19‐year‐old man with no prior morbidity presented to the emergency unit with a 2‐day history of chest pain, dry cough, and febrile sensation. One day before the onset of symptoms, he was using a liquid‐in‐glass mercury thermometer that accidentally broke and spilled mercury across the left axillary and pectoral region, after which he took a hot shower.

On admission, his blood pressure was 110/80 mmHg, temperature 38.3°C, heart rate 116 beats/min, respiratory rate 28 breaths/min, and O_2_ saturation 91% at room temperature. Laboratory testing showed a white blood cell count of 15 600 cells/μL (normal: 3.500‐10.500/μL), C‐reactive protein concentration of 123.3 mg/L (normal: <5.0 mg/L), creatinine concentration of 0.65 mg/dL, and no proteinuria.

**Figure 1:**
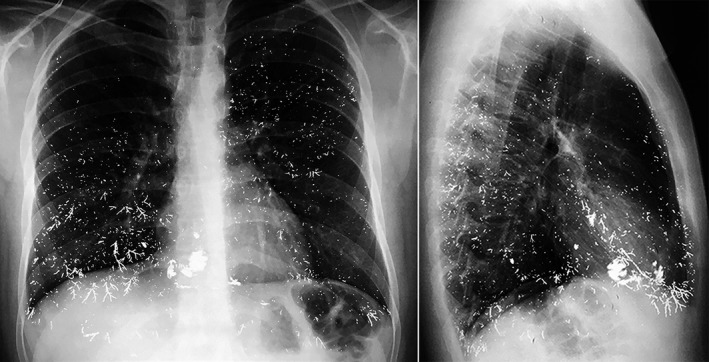
Anterior‐posterior and lateral chest X‐ray shows multiple images of metallic density in both lung fields. Some of these followed the anatomy of the bronchial tree

**Figure 2:**
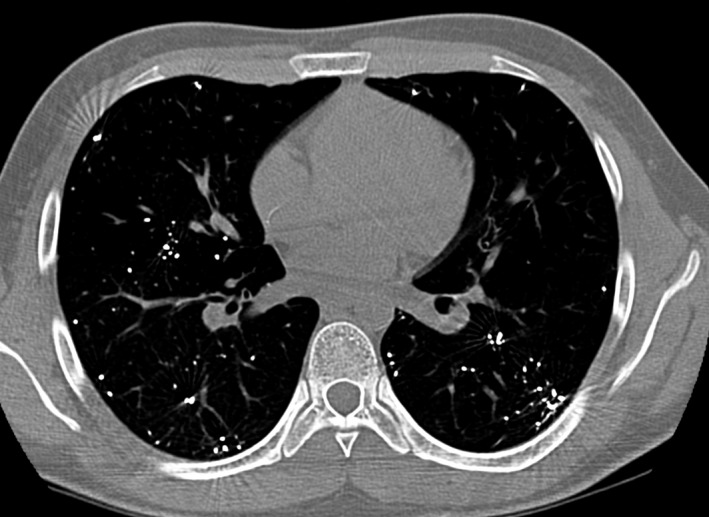
Coronal thoracic computed tomography shows the presence of multiple images of radiopaque material in both lung fields

A chest X‐ray showed multiple images of metallic density that were predominantly bibasal, some of which followed the anatomy of the bronchial tree (Figure 1). Computed tomography of the chest confirmed the radiological findings (Figure 2). His urinary mercury concentration was found to be 172 μ/g (normal: 11.50‐36.50 μ/g).

The patient was kept in the hospital under strict supervision and was provided supportive therapy according to guidelines.^9^ He progressed satisfactorily, his respiratory symptoms disappeared, and the inflammatory activity indexes normalized. After 10 days, the patient was asymptomatic and was discharged.

## 3 DISCUSSION

Elemental mercury is the only metal that exists in liquid form at room temperature.^9^ It can vaporize at room temperature,^1^, ^2^ and its tendency to do so increases with heat, which can lead to mercury poisoning.^3^, ^6^ Inhaled elemental mercury vapor is 70%‐80% absorbed by the lungs through the alveolar‐capillary membrane, which represents the main route for systemic toxicity,^10^ whereas absorption through the digestive tract^11^ and intact skin^12^, ^13^ is insignificant. Elemental mercury intoxication can be caused by accidental exposure to the mercury contained in medical appliances, such as through a broken sphygmomanometer or thermometer.^6^ In our patient, the accidental rupture of a liquid‐in‐glass mercury thermometer caused elemental mercury to spill over his skin.

Although a mercury thermometer contains about 500‐700 mg of elemental mercury, which is virtually nontoxic,^6^ significant exposure may occur through acute inhalation, which can cause acute pneumonia, adult respiratory distress syndrome, and death.^7^, ^8^ In this patient, it is probable that the hot shower immediately after exposure increased the volatility of the mercury spilled over the skin and, therefore, the amount of mercury inhaled.^3^, ^4^

The clinical picture of mercury vapor poisoning has three phases. The initial phase, during the first 1‐3 days after exposure, manifests as a flu‐like illness that includes fever, dry cough, dyspnea, and chest pain^14^, ^15^ as experienced by this patient. The intermediate phase may be accompanied by severe multiorgan symptoms, and the last phase may involve symptoms of the central nervous system.^1^, ^15^

The diagnosis of mercury poisoning in this patient was based on the clinical picture that followed the accidental exposure to mercury, the presence of metallic density distributed in both lungs as shown by chest X‐ray (Figure 1), computed tomography (Figure 2), and elevated mercury level in the urine. In some patients with elemental mercury intoxication, the symptoms may resolve spontaneously, as occurred in this patient, or may evolve toward respiratory distress, respiratory failure, and death.^7^, ^8^, ^16^ According to guidelines for out‐of‐hospital management of elemental mercury exposure, patients with symptoms such as cough, dyspnea, and chest pain should be referred immediately to an emergency department for evaluation.^6^ Asymptomatic patients with brief, unintentional, low‐dose vapor exposure can be observed at home.^6^ Although chelation therapy has been shown to reduce the serum mercury concentration, this cannot reverse lung damage.^8^, ^17^

This case report highlights the fact that, although the manufacture of glass mercury thermometers is now prohibited, they are still present in daily life. Physicians should be able to recognize the clinical manifestations of mercury poisoning caused by accidental exposure and know the appropriate treatment for it.

## CONFLICT OF INTEREST

None declared.

## AUTHORSHIP

RD‐N: involved in concept/design, data analysis/interpretation, drafting the article, critical revision of the article, and approval of the article. JC: performed data collection, critical revision of the article, and approval of the article. JP: carried out critical revision of the article and approval of the article.
